# Biopharmaceutical Assessment of Dexamethasone Acetate-Based Hydrogels Combining Hydroxypropyl Cyclodextrins and Polysaccharides for Ocular Delivery

**DOI:** 10.3390/pharmaceutics12080717

**Published:** 2020-07-30

**Authors:** Roseline Mazet, Xurxo García-Otero, Luc Choisnard, Denis Wouessidjewe, Vincent Verdoot, Frédéric Bossard, Victoria Díaz-Tomé, Véronique Blanc-Marquis, Francisco-Javier Otero-Espinar, Anxo Fernandez-Ferreiro, Annabelle Gèze

**Affiliations:** 1 University Grenoble Alpes, CNRS, DPM, 38000 Grenoble, France; rmazet@chu-grenoble.fr (R.M.); luc.choisnard@univ-grenoble-alpes.fr (L.C.); denis.wouessi@univ-grenoble-alpes.fr (D.W.); veronique.blanc-marquis@univ-grenoble-alpes.fr (V.B.-M.); 2 Pharmacy Unit, Grenoble University Hospital, 38000 Grenoble, France; 3 Department of Pharmacology, Pharmacy, Pharmaceutical Technology and Industrial Pharmacy Institute, Faculty of Pharmacy, University of Santiago de Compostela (USC), 15782 Santiago de Compostela, Spain; xurxo.garcia.otero@gmail.com (X.G.-O.); victoriadiaztome@gmail.com (V.D.-T.); francisco.otero@usc.es (F.-J.O.-E.); 4 Molecular Imaging Group, Health Research Institute of Santiago de Compostela (IDIS), 15706 Santiago de Compostela, Spain; 5 University Grenoble Alpes, CNRS, Grenoble INP, LRP, 38000 Grenoble, France; vincent.verdoot@univ-grenoble-alpes.fr (V.V.); frederic.bossard@univ-grenoble-alpes.fr (F.B.); 6 Clinical Pharmacology Group, Health Research Institute of Santiago de Compostela (IDIS), 15706 Santiago de Compostela, Spain; 7 Pharmacy Department, Clinical University Hospital Santiago de Compostela (SERGAS), 15706 Santiago de Compostela, Spain

**Keywords:** dexamethasone acetate, cyclodextrins, eye drops, hydrogels, rheology, cytotoxicity studies, transcorneal permeation, radiolabeled ocular biopermanence

## Abstract

We previously developed two optimized formulations of dexamethasone acetate (DXMa) hydrogels by means of special cubic mixture designs for topical ocular administration. These gels were elaborated with hydroxypropyl-β-CD (HPβCD) and hydroxypropyl-γ-CD (HPγCD) and commercial hydrogels in order to enhance DXMa water solubility and finally DXMa’s ocular bioavailability and transcorneal penetration. The main objective of this study was to characterize them and to evaluate in vitro, ex vivo, and in vivo their safety, biopermanence, and transcorneal permeation. Gels A and B are Newtonian fluids and display a viscosity of 13.2 mPa.s and 18.6 mPa.s, respectively, which increases their ocular retention, according to the in vivo biopermanence study by PET/CT. These hydrogels could act as corneal absorption promoters as they allow a higher transcorneal permeation of DXMa through porcine excised cornea, compared to DEXAFREE^®^ and MAXIDEX^®^. Cytotoxicity assays showed no cytotoxic effects on human primary corneal epithelial cells (HCE). Furthermore, Gel B is clearly safe for the eye, but the effect of Gel A on the human eye cannot be predicted. Both gels were also stable 12 months at 25 °C after sterilization by filtration. These results demonstrate that the developed formulations present a high potential for the topical ocular administration of dexamethasone acetate.

## 1. Introduction

Dexamethasone (DXM) is one of the most prescribed anti-inflammatory drug in the treatment of acute and chronic eye inflammation due to its high potency and effectiveness [1]. DXM acts by binding with the corticosteroid receptors present in the human trabecular meshwork cells and inhibits phospholipase-A2 and thus prostaglandins synthesis. DXM eye drops, as MAXIDEX^®^ 1 mg/mL DXM (Novartis Pharma, Rueil-Malmaison, France) and DEXAFREE^®^ 1 mg/mL DXM phosphate (Laboratoires Théa, Clermont-Ferrand, France) are effective in treating postoperative inflammation, keratitis, uveitis [2], and prevention of corneal graft rejection [3]. Despite the many advantages offered by this route of administration, these marketed formulations present a major disadvantage by requiring frequent administrations (up to six times/day) [2]. This is due to the presence of various anatomical and physiological barriers, which leads to a poor bioavailability of the ophthalmic drugs; only 1–5% of drug instilled reaches in aqueous humor [4].

In order to enhance DXM bioavailability, the lipophilic derivative DXM acetate (DXMa), currently unavailable for ophthalmic topical use, could be very interesting. Indeed, DXMa has shown to readily permeate the cornea and be hydrolyzed into DXM during absorption [5]. Furthermore, Leibowitz et al. demonstrated that the acetate form was more effective compared to the phosphate derivative in suppressing inflammation in the cornea. This therapeutic effect was not associated with a greater propensity to increase intraocular pressure, one of the most frequent side effects of glucocorticoids [6].

Furthermore, for the topical administration of DXMa into the eyes, we previously developed, by means of experimental designs, two optimized formulations based on HPβCD or HPγCD/DXMa solutions and marketed gels, with the aim of increasing DXMa bioavailability and reducing instillation frequency. HPβCD or HPγCD have considerably enhanced DXMa solubility in water, 500, and 1550-fold [7]. CELLUVISC^®^ (sodium carboxymethylcellulose) and VISMED^®^ (sodium hyaluronate) have both been used as an artificial tear in order to stabilize the tear film on the ocular surface [8]. Carboxymethylcellulose (CMC) and sodium hyaluronic (NaHA) present great advantages to be mucoadhesive, biodegradable, and biocompatible [9]. These properties exhibit an enhancement of the precorneal residence time and a reduction in the nasolacrymal drainage due to increased viscosity [10]. In addition, NaHA has been shown to modulate the inflammation response of the ocular surface in dry eye syndrome [11].

In the present study, the optimized formulations were characterized. The ocular in vitro cytotoxicity and mucoadhesion properties were evaluated as well as ex vivo transcorneal permeation of DXMa. Furthermore, in vivo precorneal drug kinetics were investigated by radiolabeling with ^18^F-FDG in order to show the benefits of the newly designed formulations.

## 2. Materials and Methods

### 2.1. Materials

DXMa was purchased from LA COOPER (Melun, France). Hydroxypropyl-γ-cyclodextrin (HPγCD, W8HP, DS = 0.6, and Mw = 1576 Da) was a kind gift from ASHLAND (Schaffhausen, Switzerland) and hydroxypropyl-β-cyclodextrin (HPβCD, KLEPTOSE DS = 0.63 and Mw = 1391 Da) was obtained from ROQUETTE (Lestrem, France). CELLUVISC^®^ (sodium carboxymethylcellulose) and VISMED^®^ (sodium hyaluronate) are marketed gels used for the treatment of dry eye syndrome. DEXAFREE^®^ (DXM sodium phosphate 1% solution eye drops), MAXIDEX^®^ (DXM 0.1% suspension eye drops) and BSS^®^ (Alcon Laboratories, Rueil-Malmaison, France) are human authorized ocular medicines. Normal human primary corneal epithelial cells (ATCC PCS 700-010), medium (ATCC PCS-700-030), growth kit (ATCC PCS-700-040), PBS (ATCC 30-2200), trypsin EDTA (ATCC PCS-999-003 and 005), and antibiotics (gentamicin, streptomycin, and amphotericin BATC PCS-999-002) were obtained from LGC standard - ATCC^®^ (Molsheim, France). Thioglycollate with resazurine medium and Tryptic soy broth were obtained from BIOMERIEUX (Craponne, France). ALAMARBLUE^®^ was purchased from BIO-RAD (Marnes-la-Coquette, France) and DMSO from SIGMA-ALDRICH (Lyon, France). Purified water was prepared by DIRECT-Q^®^3UV water purifier (MILLIPORE, Molsheim, France). All other solvents and chemicals were of HPLC and analytical grade, respectively.

### 2.2. Methods

#### 2.2.1. Gel Composition

The composition of optimized mixed Gels A and B were obtained by means of experimental design, as previously described [7] (Table 1).

In a first step, the cyclodextrin derivative was introduced in sterile water (600 mg/mL) and agitated at room temperature. Then, the DXMa powder was added to the cyclodextrin solution. After complete drug dissolution, the DXMa (10 mg/mL)/HPβCD (600 mg/mL) and DXMa (30 mg/mL)/HPγCD (600 mg/mL) were obtained. In a second step, CELLUVISC^®^ (sodium carboxymethylcellulose) and/or VISMED^®^ (sodium hyaluronate) were added to the DXMa/CD aqueous solutions in order to obtain the final Gels A and B, according to the ratio described in Table 1.

#### 2.2.2. Sterilization Step

Two different methods were investigated with Gels A and B (i.e., autoclaving, SANO CLAV from ADOLF WOLF, Überkingen, Germany) at 121 °C during 20 min or double sterilizing filtration (CME or PVDF 0.22 µm filter, ROTH, Karlsruhe, Germany) and conditioned in sterile vials under laminar air flow of an ISO 4.8 microbiological safety cabinet.

#### 2.2.3. Physicochemical Characterizations

##### Drug Quantification

The drug quantification methodology was adapted from that previously reported [7,12] and validated in DXMa concentrations according to ICH Q2 (R1) guidelines in order to evaluate specificity, linearity, repeatability, intermediate fidelity, and limit of detection (LOD) and limit of quantification (LOQ) [13]. Quantitative determination were performed on a reversed-phase, high-performance liquid chromatographic (HPLC) component system LC 2010 AHT (SHIMADZU, Kyoto, Japan) consisting of a pump with degasser, an autosampler, a UV–VIS detector, and a column XTERRA^®^MS C8, 5 µm particles, and 150 × 4.6 mm with a C8 cartridge. The mobile phase made of methanol:water (70:30 v/v) was set at the rate of 1.2 mL/min. The column was thermo-regulated at 25 °C. The detection wavelength was set up at 240 nm.

##### Method Validation

The method was validated according to the International Conference on Harmonization (ICH) guideline Q2 (R1) “Validation of Analytical Procedures” [13].

##### Linearity and Accuracy Studies

Five standard samples at different concentration values were prepared using 0.1 mg/mL DXMa as a solution stock. Table 2 contains the different sample concentration levels for Gels A and B.

These calibration levels were analyzed twice a day during three days [14]. The peak area was plotted against the concentration at each level and a calibration curve was generated by a linear least square regression analysis by checking the pre-required assumptions.

##### Specificity

The specificity of the developed method was first established by verifying that all the components of gels were separated from the DXMa chromatographic peak. In complement, to exclude potential interference of degradation products with DXMa quantification, DXMa 1 mg/mL solutions, Gel A, and Gel B were subjected to forced degradation conditions, according to SFSTP guidelines [15]: 0.5 N hydrochloric acid or sodium hydroxide, at 80 °C for 60 min, in 3% hydrogen peroxide at 80 °C for 4 h and under visible and ultraviolet light for 6 h.

##### Precision

Intra-day (repeatability) and inter-day (intermediate) precision assays were determined by preparing a model solution at a 100% concentration level (70 µg/mL for Gel A and 200 µg/mL for Gel B). Each solution was analyzed six times a day for three days.

Limit of detection (LOD) and limit of quantification (LOQ) were estimated from the standard deviation of the response as well as the slope, according to ICH guidelines. The estimated results were not empirically verified.

##### Rheological Measurements

Rheological characteristics of both gels were examined at various shear rates using an ARES-G2 rheometer from TA Instruments (New Castle, PA, USA) equipped with a Couette system (cup diameter 33.985 mm, upper cylinder diameter 32 mm, and APS kit) from TA Instruments (New Castle, USA). The measuring corresponded to 1.0620 according to ISO 3219. The gap length was 2 mm and the sample volume of >5.2 mL. The temperature was controlled at 35 °C by a Peltier jaquette.

The steady-state flow experiments were performed in the range of 0.11 to 100 s^−1^. The frequency sweep method was performed between 0.1 Hz and 10 Hz, with a shear strain of 10% for both formulations, while the table of shear rate method was performed by increasing the shear rate from 0.1 to 100 s^−1^, at 35 °C. The shear stress was measured by this method and the apparent viscosity was calculated by dividing the shear stress by the shear rate. An oscillatory amplitude sweep and frequency testing were performed using this equipment.

The amplitude sweep conditions used were shear strain between 0.1% and 100% with the frequency of 0.1 Hz. Amplitude tests showed that 10% deformation corresponded to a value in the linear range. In the frequency testing, the frequency range used was between 0.1–10 Hz with a shear strain of 10%.

#### 2.2.4. Mucoadhesion

In this study, mucin was rehydrated with water by gentle stirring until complete dissolution to yield a dispersion of 10% (w/w) at 20–25 °C. The mucoadhesion was evaluated by the effect of mucin on zeta potential (ZP) values of Gel A ± mucin (1:1), Gel B ± mucin (1:1). A volume of 40 µL of Gel A, Gel B, and mucin were diluted in either 2 mL of sterile purified water [16,17,18]. The ZP values of the different mixtures were measured using a Zetasizer Nanoseries Nano ZS (Malvern Instruments, Malvern, UK) at 35 °C. All the experiments were done in triplicate.

#### 2.2.5. Cytotoxicity Studies

Two different cellular toxicity assays were used based on cell viability in relation to mitochondrial enzymes [19] (i.e., the methylthiazolyldiphenyl-tetrazolium bromide conversion (MTT) and ALAMAR BLUE^®^ assays). The experiments were performed using normal human primary corneal epithelial cells (HCEC) obtained from ATCC^®^ and maintained in an incubator (37 °C and 5% CO_2_ saturation). HCEC were kept in corneal epithelial cell growth culture medium with gentamicin and amphotericin B, without fetal bovine serum. All experiments were performed in between steps 4 and 8. Three thousand cells per well (96 wells per plates) were incubated for 24 h at 37 °C and 5% CO_2_ in order to have between 80 and 90% of cell confluence, according to ATCC^®^ protocol. Subsequently, during the MTT assay, the original culture medium was aspirated and different concentrations (25 μL/200 µL, and 0.25 µL/200 µL) of different formulations: Gels A and B with or without DXMa, HPβCD (600 mg/mL) and HPγCD (600 mg/mL) aqueous solutions, DEXAFREE^®^ eye drop solution, and MAXIDEX^®^ eye drop suspension were added to different wells and incubated for 30 min and 2 h. Each concentration was tested in three individual wells. After 30 min, 2 h, and 24 h, the supernatant was removed and 200 µL of MTT solution (5 mg/mL in PBS and then diluted to 1/10 in complete medium) was added to each well and then incubated for 3 h at 37 °C to allow the formation of formazan crystals. The medium was then removed, and blue formazan was eluted from cells by 200 µL of DMSO. The plates were shaken in order to solubilize the crystals of formazan. The liquid was aspirated to another new 96-wells plate and measured directly at 590 nm with Clariostar (BMG Labtech, Champigny sur Marne, France). Each plate was duplicated. The cell viability values were compared using a well-known t-test procedure with α = 5%. Additionally, the ALAMAR BLUE^®^ was performed after 2 h of incubation at 37 °C, 5% CO_2_, with the IC50 concentrations as determined by the MTT assay. A total of 20 µL of ALAMAR BLUE^®^ reagent were added in each well before 2 h of incubation at 37 °C, 5% CO_2_. Fluorescence was measured with an excitation wavelength at 530–560 nm and emission wavelength at 590 nm with Clariostar (BMG Labtech, Champigny sur Marne, France). Each plate was duplicated.

The % of reduction of ALAMAR BLUE^®^ can be calculated by Equation (1):

(1) $$\begin{array}{l} \%\;\mathrm{Reduction}\;\\ =\frac{\left(\mathrm{Experimental}\;\mathrm{RFU}\;\mathrm{value}\right)-\left(\mathrm{Negative}\;\mathrm{control}\;\mathrm{RFU}\;\mathrm{value}\right)}{\left(100\%\;\mathrm{reduced}\;\mathrm{positive}\;\mathrm{control}\;\mathrm{RFU}\;\mathrm{value}\right)-\left(\mathrm{Negative}\;\mathrm{control}\;\mathrm{RFU}\;\mathrm{value}\right)}\times 100, \end{array}$$

#### 2.2.6. Ex Vivo Evaluation of the Corneal Permeation

The transcorneal permeation experiment was performed for Gels A and B, DEXAFREE^®^, and MAXIDEX^®^ using Franz diffusion cells with an available diffusion area of 1.131 cm^2^. The porcine corneas were recovered from the slaughterhouse in accordance with ethical regulations. The corneas were removed and then mounted onto diffusion cells, with the epithelial layer exposed to the donor chamber. The latter was filled with 0.4 g of each ophthalmic formulation; whereas the receptor chamber was filled with 13 mL artificial tear fluid Balanced Salt Solution (BSS) According to Wen et al. [20], the experiment was performed at 35 ± 1 °C in a thermostatic water bath with a moderate speed of rotation maintained for 24 h. Three corneas per formulation (n = 3) were used. A 1 mL sample was removed at predetermined time intervals (15 min, 30 min, 1 h, 2 h, and 4 h) and replaced with an equal volume of fresh medium to maintain the sink conditions. The withdrawn samples from the receptor chamber were analyzed by HPLC. The cumulative amount of drug appearing in the receptor compartment (*Qn*) was plotted as a function of time (t_n_) and calculated using Equation (2):

(2) $$Qn=V_{0\;}\left(C_{n}+\frac{V}{V_{0}}\sum_{i=1}^{n-1}C_{i}\right)={\;V}_{0}C_{n}+V\sum_{i=1}^{n-1}C_{i},$$

C_n_*:* Drug concentration at t time points (µg.mL^−1^), C_i_*:* Drug concentration at sampling points, V_0_*:* Volume of the medium in the receiving chamber, and V: sampling volume.

The corneal hydration level (% HL) was measured with a relative humidity analyzer MB45 OHAUS^®^ (Parsippany, NJ, USA).

#### 2.2.7. In Vivo Evaluation of the Residence Time on the Ocular Surface

In vivo studies were carried out on male Sprague-Dawley rats with an average weight of 250 g supplied by the animal facility at University of Santiago de Compostela (Spain). The animals were treated according to the laboratory guidelines [21]. The experiments were approved by the Galician Network Committee for Ethics Research following the Spanish and European Union (EU) rules (86/609/CEE, 2003/65/CE, 2010/63/EU, RD 1201/2005 and RD53/2013)The project identification code was IDIS12072017, 12/07/2017 was approved by the Health Research Institute of Santiago de Compostela institutional review board. The animals were kept in individual cages at controlled conditions of temperature and humidity (22 °C and 60%) with free access to water and food, with day–night cycles regulated by artificial light. Each component of the optimized formulations, in other words, CELLUVISC^®^, VISMED^®^, DXMa (10 mg/g)/HPβCD (600 mg/mL), and DXMa (30 mg/g)/HPγCD (600 mg/mL) aqueous solutions; Gel A and Gel B were radiolabeled by incorporating 100 µL ^18^F-fluorodeoxyglucose (^18^F-FDG) in a volume of 1 mL of either hydrogel or cyclodextrin based aqueous solution until homogenization, according to the methodology followed in previous studies [22]. Randomly taken samples from each labeled component were measured using a high-precision dose calibrator (Atomlab 500, Biodex Medical System, Inc., New-York, NY, USA) in order to control radiotracer uniformity. Positron emission tomography and computerized tomography (PET/CT) images were acquired using the Albira PET/CT Preclinical Imaging System (Bruker Biospin, Woodbridge, CT, USA). The anesthetized animals were positioned into the imaging bed and 7.5 μL of each formulation labeled with^18^F-FDG was instilled into the conjunctival sac of eye using a micropipette. The administered radioactivity was 0.35 ± 0.08 MBq. Therefore, the ^18^F-FDG labeled component (CELLUVISC^®^, VISMED^®^), DXMa (10 mg/g)/HPβCD (600 mg/mL), and DXMa (20 mg/g)/HPγCD (600 mg/mL) aqueous solutions as well as the ^18^F-FDG labeled optimized gels (A or B) were tested. Immediately after administration, static PET frames of 10 min were acquired and the animal was awakened. Then, single frames of 10 min at 0.5, 1, 2, 3, and 5 h after instillation were acquired, anesthetizing the animal 5 min before obtaining the images and then waking it up. Three eyes of three animals were tested for each formulation. The results were corrected to radioactive decay. Graphical representations of radioactivity versus time were obtained. The % remaining formulations on ocular surface was calculated as the ratio of radioactivity at time t in ocular surface/initial radioactivity. The fitting of the remaining formulation versus time to a monoexponential decay equation using a single compartmental model was performed using pKSolver [23]. A non-compartmental analysis was also performed calculating the mean residence time (MRT) and the total area under the curve (AUC) of the remaining formulations (%) versus time. All data were expressed as mean value ± standard deviation (SD). Statistical analyses were performed using one-way ANOVA test, and the level of significance was set at 5%.

#### 2.2.8. Stability Studies

Both formulations were prepared using sterile water, HPβCD, HPγCD, DXMa, VISMED^®^, and CELLUVISC^®^ under laminar air flow of an ISO 4.8 microbiological safety cabinet. A total of 2 mL of each gel was conditioned into a 5 mL glass vial previously autoclaved, closed with a polypropylene cap, and sealed with an aluminum ring. Two batches of each gel were prepared and submitted to a double filtration with PVDF 0.22 µm filters.

The stability of each gel was studied in unopened multidose eyedroppers for 12 months at 25 °C in a climate chamber (BINDER GmbH, Tuttlingen, Germany). Four units per formulation were subjected to visual inspection, DXMa quantification, sterility, osmolality, and pH measurements at times 0, 14, and 30 days, 2, 6, 9, and 12 months. More precisely, for each unit, color and aspect were checked. DXMa was quantified by HPLC and the degradation product sought using a stability indicating method [13]. Gels A and B were previously diluted by 1/100 with the mobile phase. Osmolality was measured using a 2020 freezing point osmometer (Advanced Instruments, Norwood, United States). pH measurements were made with a SevenMulti^®^ pH-meter with an InLab electrode (Mettler-Toledo, Viroflay, France). The sterility test was carried out according to the European Pharmacopeia sterility assay (2.6.1) [24]. Briefly, the multidose eyedroppers were opened under the laminar air flow of an ISO 4.8 microbiological safety cabinet and the content was divided into two equal parts, each transferred in a fluid thioglycollate with resazurine medium and Tryptic soy broth and incubated at 30–35 °C and 20–25 °C, respectively for 14 days. The culture medium was examined every day.

## 3. Results and Discussion

### 3.1. Drug Quantification Before and After Sterilization

Pure DXMa and DXMa formulated in Gels A and B presented a retention time of 3.2 ± 0.2 min and their chromatograms are presented in Supplementary Materials (Figure S1).

#### Method Validation Studies

The RP-HPLC method used to analyze the DXMa in Gels A and B was validated according to current ICH Q2(R1) [13]. The performed validation tests proved the suitability of the method for its intended purposes. Validation tests including specificity, linearity and range parameter, accuracy, precision, LOQ, and LOD. Original validation data are reported in the Supplementary Materials.

### 3.2. Sterilization Step

Two different methods were investigated with Gels A and B (i.e., autoclaving, SANO CLAV from ADOLF WOLF, Überkingen, Germany) at 121 °C during 20 min or double sterilizing filtration (CME or PVDF 0.22 µm filter, ROTH, Karlsruhe, Germany). The sterile filtered product was packaged in sterile vials under laminar air flow of an ISO 4.8 microbiological safety cabinet. The choice of the sterilization steps is primordial and was evaluated in terms of change in chromatographic profile and in % of drug loss. As seen in Supplementary Materials Figures S5 and S6, a peak of degradation product appeared and the DXMa peak was reduced. Therefore it excludes autoclaving as a sterilization method of DMXa. DXMa seems to be heat labile, and a similar result is reported in the literature for dexamethasone sodium phosphate [25].

The CME filters were discarded because they led after filtration to a loss of 12.9 ± 0.5% DXMa with Gel A and 5.3 ± 0.3% with Gel B, while the filter PVDF resulted in only a loss of 0.6 ± 0.02% DXMa with Gel A and 0.4 ± 0.02% with Gel B. The PVDF filters were therefore retained and were confirmed by demonstrating the repeatability of the sterilization step without a great loss of DXMa. Indeed, six samples of each gel were prepared and DXMa was quantified by HPLC before and after the double filtration steps with PVDF 0.22 µm filters. The relative percentage of standard deviation (RSD) of drug quantification was calculated from these quantifications. For both formulations, the drug loss was <0.3% and the repeatability RSD values were 0.96% (Gel A) and 0.95% (Gel B). The RSD (%) values were found to be <1%, which were considered acceptable.

### 3.3. Rheological Measurements

**Figure 1:**
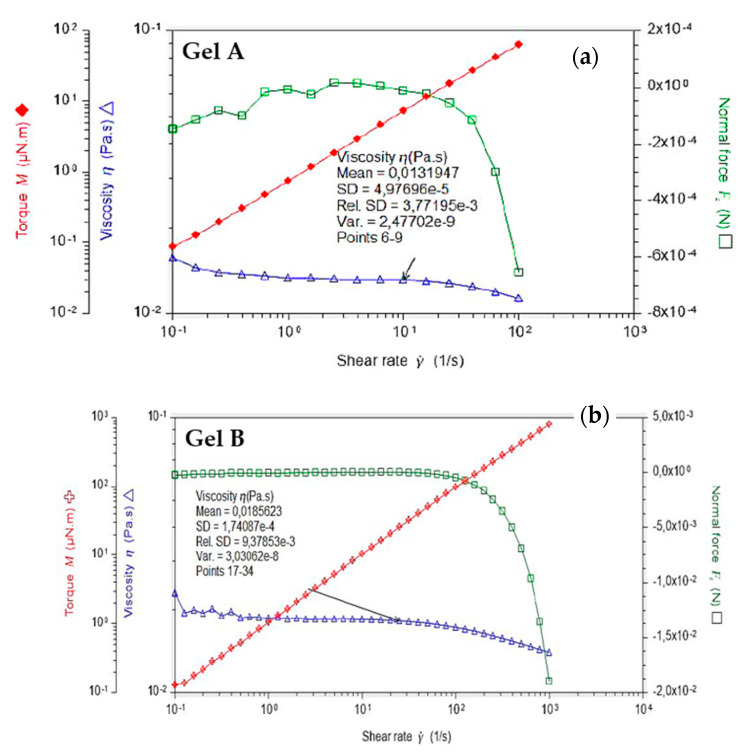
Dynamic viscosity of (**a**) Gel A and (**b**) Gel B performed in the range of 0.11 to 100 s^−1^ at 35 °C.

The administration of an ophthalmic formulation should not influence the pseudoplastic nature of precorneal film, or the influence should be negligible. Figure 1a,b present the dynamic viscosity of each formulation as a function of shear rate (0.11–100 s^−1^) at 35 °C, measuring five points per decade and with 20 s equilibration time. The both formulations exhibited Newtonian behavior. At shear gradients greater than 70–80 s^−1^, centrifugal forces and turbulences come into play, which results in a fall in axial force. The apparent rheofluidifying behavior past 100 s^−1^ is therefore an artifact caused by these centrifugal forces. For shear rates of less than 1 s^−1^, the crust formed by the eye drops when drying opposes a resistance to the rotational movement of the geometry, which is no longer negligible compared to the measured torque, which explains the slight rise in the curve between 0.1 and 1 s^−1^. Below 0.3 s^−1^, this crust makes measurements imprecise and so between 0.3 and 100 s^−1^, Gels A and B present a Newtonian behavior, Gel A displays a viscosity of 13.2 mPa s ± 10%, and Gel B a viscosity of 18.6 mPa s ± 10% (Figure 1). As demonstrated by Zaki et al., the retention on eye surface began to increase only after a viscosity exceeding a critical value of about 10 mPa s [26].

Although increasing fluid viscosity improves the residence time, it may also cause discomfort and damage to ocular epithelia due to an increase in the shear stresses during blinking. Carboxymethylcellulose and sodium hyaluronate are well known for their viscosifying properties. Furthermore, sodium hyaluronate, present in Gel A, Gel B, and VISMED^®^, is a shear thinning fluid. Sodium hyaluronate should contribute to enhance viscosity while avoiding excessive stresses during blinking [27]. Additionally, these viscosities, lower than 30 mPa.s, are well tolerated by patients because it does not lead to blurred vision and foreign body sensation, resulting in a faster elimination due to reflex tears and blinks [28].

Before oscillation frequency sweep, an amplitude sweep test was performed to define the fluid’s linear-viscoelastic region (LVER), and 10% at least for both formulations. Indeed, for Gel A, the amplitude sweep test performed at 1 Hz between 0.1 and 100% did not indicate any output of the linear domain. For Gel B, the oscillation measured between 0.1 and 100% of deformation did not show any upper limit and so, caution should be used to avoid not being below 1.5% deformation with this rheometer (Supplementary Materials Figure S7). At 0.1 Hz, the storage module was negligible, which explains why some points are missing on the graphs (negative values cannot be displayed on a logarithmic scale). For both at low amplitudes, the signal becomes lower than the sensitivity of the material (0.05 μNm).

**Figure 2:**
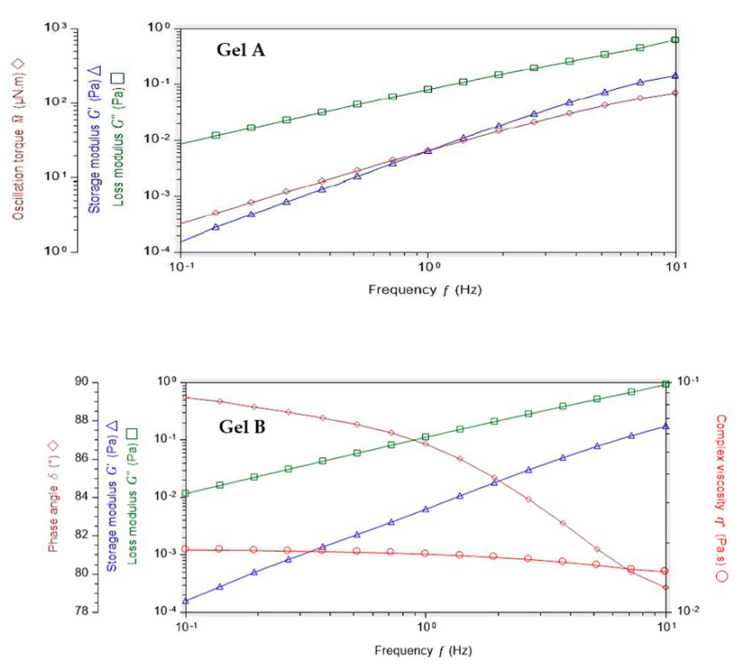
Oscillation frequency performed with Gels A and B between 0.1–10 Hz with a shear strain of 10% at 35 °C.

With these results, Gels A and B can be further characterized using a frequency sweep, proving more information about the effect of colloidal forces [29]. Figure 2 presents oscillation frequency performed between 0.1–10 Hz with a shear strain of 10% at 35 °C. Both formulations exhibited fluid-like mechanism spectra with G” modulus even greater than G’, being both frequency dependent.

#### Mucoadhesion

**Figure 3:**
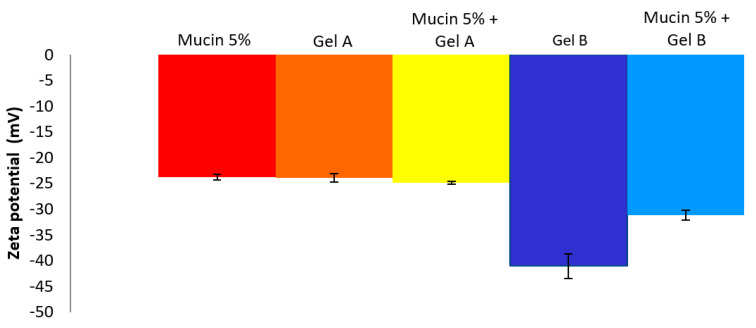
Zeta potential values (mean ± SD, n = 3) of mucin 5%, Gel A, Gel B, and mucin 5% + Gel A or Gel B.

Zeta potential (ZP) value is related to the measurement of the surface charge that a specific material possesses or acquires when suspended in a fluid. This study demonstrated that the ZP values of Gel A and Gel B are quite different. Indeed, Gel B ZP value (−41.1 ± 2.3 mV) is much more negative than Gel A (−23.9 ± 0.7 mV) (Figure 3). These negative values are in accordance with the anionic nature of the hyaluronic acid due to the presence of carboxylic groups. HA is present in VISMED^®^, Gel A, and Gel B. The mucins also presented a negative ZP value due to their carboxyl and sulfate groups. The obtained value was quite different from the one described in the literature, which was approximately −10 mV [16]. This difference could be explained by a different degree of hydration [30]. When the mucin 5% (w/v) suspension was added to Gel B, an increase of the negative charge was observed, showing the reduction in electrostatic repulsion, and indirectly an interaction of the vehicle with the mucins [31].

### 3.4. Cytotoxicity Studies

#### 3.4.1. MTT

**Figure 4:**
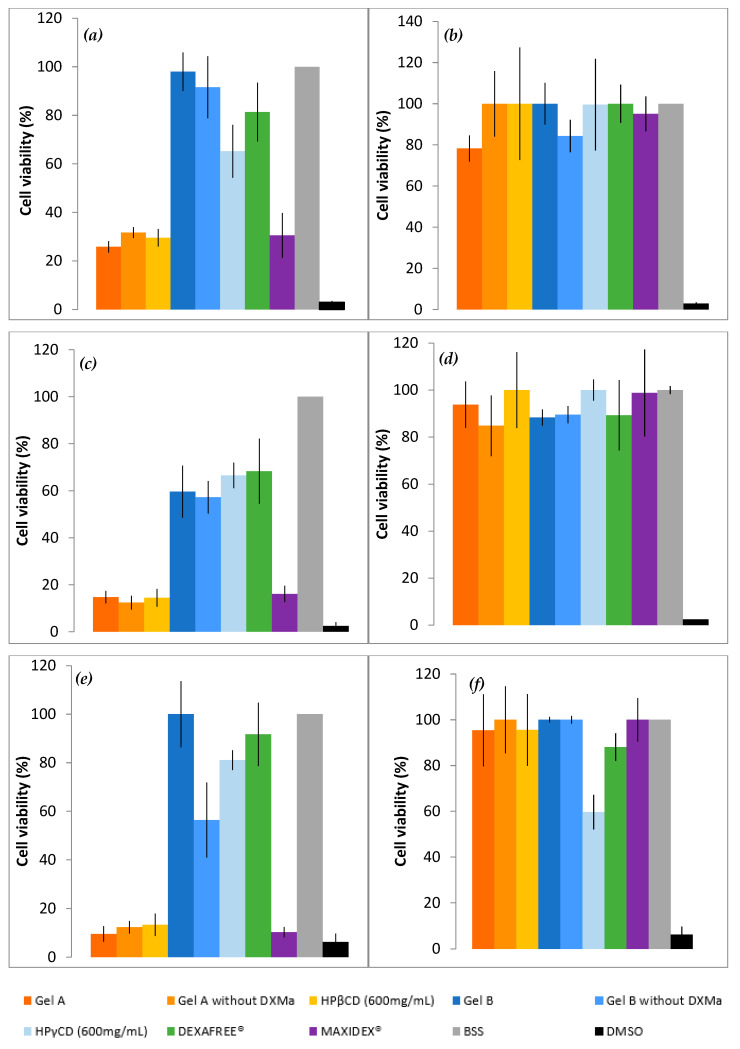
Cell viability of Gels A and B with or without DXMa, HPβCD (600 mg/mL) and HPγCD (600 mg/mL) aqueous solutions, DEXAFREE^®^, and MAXIDEX^®^. (**a**) 5% concentration during 30 min, (**b**) 0.05% during 30 min, (**c**) 5% during 2 h, (**d**) 0.05% during 2 h, (**e**) 5% during 24 h, (**f**) 0.05% during 24 h.

To evaluate in vitro cell toxicity of Gels A and B with or without DXMa, HPβCD (600 mg/mL) and HPγCD (600 mg/mL) aqueous solutions, DEXAFREE^®^ and MAXIDEX^®^, HCE cells grown in the presence of each formulation were evaluated by quantitative determination of living cells, after 30 min, 2 h, and 24 h at 5 and 0.05% concentration (Figure 4). The results were analyzed according to the Organization for Economic Co-operation and Development (OECD) guidelines for short time exposure in vitro test method [32] (Table 3).

As shown in Figure 4, Gel B showed an acceptable level of cytotoxicity to HCE cells and is considered rather well tolerated by HCEC with a cell viability higher than 70% at 5% and 0.05% at 30 min (p value < 0.001) and 24 h. In addition, DEXAFREE^®^ presented a similar cytotoxicity profile after 30 min and 24 h (p value = 0.035). In contrast, Gel A was classified in the non-predictable category since the cell viability was lower than 70% at 5% (p-value < 0.001) and higher than 70% at 0.05% (p-value = 0.022) at 30 min and 24 h. A similar cytotoxicity profile was observed in the case of the reference suspension MAXIDEX^®^. A cell viability lower than 70% at 5% (p-value = 0.01) and higher than 70% at 0.05% (p-value = 0.04) at 30 min and 24 h. The cytotoxic effect of Gel A was time and concentration dependent and seems to be caused mainly by the HPβCD (600 mg/mL) aqueous solution. Indeed, the cell viability of Gel A with or without DXMa and HPβCD (600 mg/mL) aqueous solution were relatively similar at each time and each concentration with values decreasing from around 30%, 15%, and 10% at 30 min, 2 h, and 24 h, respectively. Furthermore, each CD derivative at the concentration of 600 mg/mL presents a cytotoxic effect more or less pronounced. These observations could be attributed to the known capacity of CDs to extract and solubilize cholesterol from membranes, potentially causing destruction of phospholipid bilayers [33]. One can note that in the present study, HPγCD had a much less pronounced effect than HPβCD, showing a cell viability higher than 65%, against lower than 30% for HPβCD (p-value < 0.001 at 30 min). Moreover, the cytotoxicity of HPβCD is enhanced with increasing exposure time [34]. These differences may be attributed to the higher propensity of the βCD derivative to solubilize cholesterol from membranes compared to γCD [33]. Moreover, the clear decreased cytotoxicity observed in the case of Gel B may be related to the lower extent of free cavities available for complexation in the case of HPγCD for which a higher complexation efficiency value was previously described [7], allowing Gel B to be relatively safe for HCEC. Therefore, in the future, it will be possible to consider a lower concentration of HPβCD in Gel A in order to improve ocular tolerance [34], even if this means reducing the solubilized DXMa fraction.

#### 3.4.2. ALAMAR BLUE^®^ Assay

**Figure 5:**
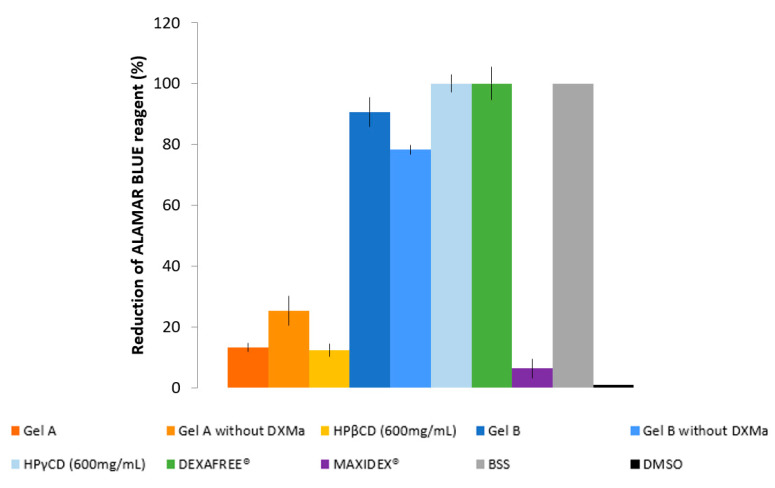
Reduction of ALAMAR BLUE^®^ reagent (%) of Gels A and B with or without DXMa, HPβCD (600 mg/mL) and HPγCD (600 mg/mL) aqueous solutions, DEXAFREE^®^, and MAXIDEX^®^.

To complete the in vitro cell biocompatibility study, the ALAMAR BLUE^®^ assay was performed by using fluorescence, which is proportional to the number of cells with metabolic activity (Figure 5). Gel B, Gel B without DXMa, and HPγCD showed acceptable levels of metabolic activity as DEXAFREE^®^, with a cell viability even >70% after 2 h of exposure. Unfortunately Gel A, Gel A without DXMa, and HPβCD (600 mg/mL) showed a low metabolic activity of <30%, which could cause serious eye damage. According to these results, we can demonstrate different biocompatibility profiles between Gel A and Gel B, probably related to the difference in biocompatibility profile between HPβCD and HPγCD. Hence, Gel B is considered as biocompatible and the formulation of Gel A might be optimized regarding the effect of HPβCD on HCEC.

### 3.5. Ex Vivo Evaluation of the Corneal Permeation

**Figure 6:**
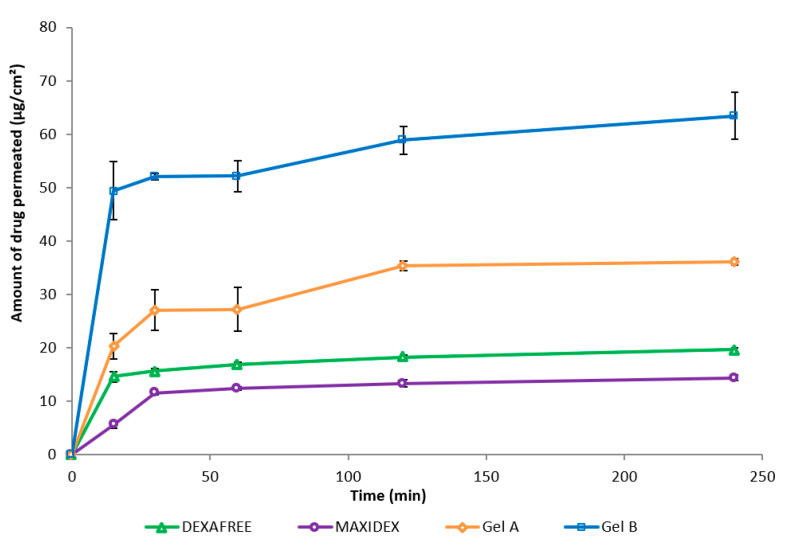
Amount of drug permeated (mean ± SD, n = 3) through the excised cornea of Gels A and B, DEXAFREE^®^, and MAXIDEX^®^ as a function of time.

Ex vivo permeation of Gel A, Gel B, DEXAFREE^®^, and MAXIDEX^®^ were evaluated using the excised porcine cornea. The amount of DXMa permeated through the excised cornea from Gel B was higher than that of the other formulations (Figure 6). With Gel B, a maximum of 71.71 µg of DXMa permeates (i.e., 0.89% amount of the drug applied) and was nearly 3.2-fold higher than DEXAFREE^®^ and 4-fold higher than MAXIDEX^®^. Gel A also presented a good corneal permeation with a maximum of 40.48 µg (i.e., 1.44% amount of the drug applied), which is 1.8-fold higher than DEXAFREE^®^ and 2.5-fold higher than MAXIDEX^®^. This suggests that both Gels A and B might be more effective than reference marketed formulations to treat corneal inflammations. Moreover, these results are associated with a good corneal hydration level between 76 and 80%.

Dexamethasone is a highly potent long acting drug requiring a far lower dosage compared to other intermediate and short acting glucocorticoids (i.e., nearly five times lower than prednisolone, methylprednisolone, and 25 times lower than hydrocortisone) to elicit a biological response [35,36]. As demonstrated by Djalilian et al., dexamethasone inhibits inflammatory cytokines in human corneal epithelial cells and fibroblast cell lines with a concentration range of 0.1 to 10 μΜ [37]. The marketed formulation DEXAFREE^®^ contains 1 mg/mL drug (i.e., 1.9 mM). As previously described, Gel B released DXMa allowing a maximum drug amount of 63.4 μg/cm^2^ to be permeated across the excised cornea. In addition, Gel A allowed a permeated drug amount of 36.07 µg/cm^2^.

Therefore, considering the normal tear volume to be about 6 to 10 μL, assuming no tear drainage and similar release behavior as observed in 13 mL of PBS, 71.71 μg and 40.48 µg of DXMa (Mw = 434.5 g/mol) in 10 μL of tears would theoretically be almost 16.6 mM and 9.3 mM, which is about 8- and 5-fold higher than the concentration provided by DEXAFREE^®^. These latter results warranted to be clinically relevant and within the therapeutic index [37].

### 3.6. In Vivo Evaluation of the Residence Time on the Ocular Surface

**Figure 7:**
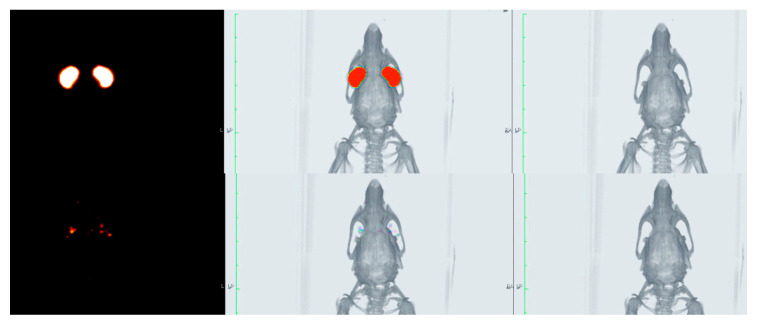
PET (**left**), CT (**right**), and fused image PET/CT (**center**) of the rat eyes immediately after administration (**top**) and 300 min post-administration (**bottom**).

**Figure 8:**
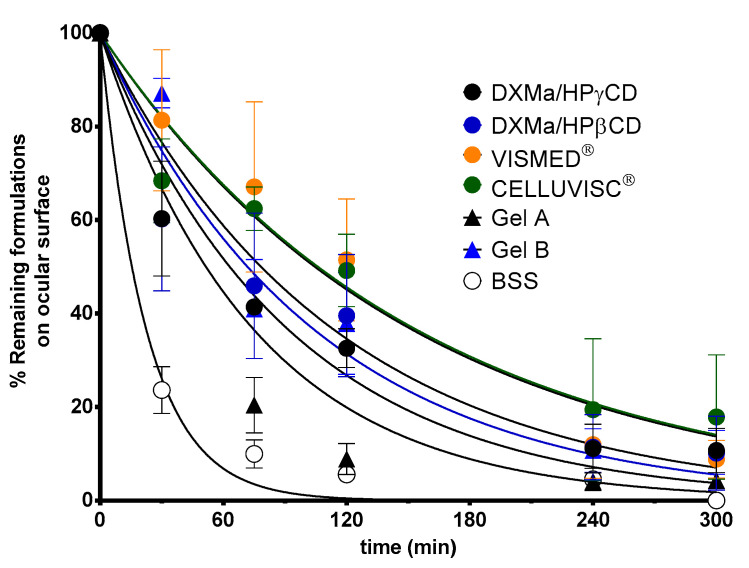
Ocular biopermanence of Gels A and B, DXMa (10 mg/mL)/HPβCD (600 mg/mL), DXMa (30 mg/mL)/HPγCD (600 mg/mL), VISMED^®^, and CELLUVISC^®^ versus BSS.

The biopermanence of Gels A and B, DXMa (10 mg/mL)/HPβCD (600 mg/mL), DXMa (30 mg/mL)/HPγCD (600 mg/mL), VISMED^®^, and CELLUVISC^®^ was characterized on the ocular surface of rats by ^18^F-FDG radiolabeling followed by radioactivity in PET over 5 h (300 min) (Figure 7). It is a non-invasive tool for pharmacokinetic studies of clearance of topical ocular drug delivery systems [22,38]. In the present study, all the formulations tested presented a higher residence than the control solution of Balanced Salt Solution (BSS), whose composition is close to tears. Indeed, in Figure 8, it can be observed that after 30 min of contact, 23% of the BSS remained in the ocular surface against 60 to 100% remaining doses for the other formulations.

These observations are in accordance with the PET data described by Luaces-Rodriguez et al. in the case of tacrolimus eye drops [39]. According to the literature, increasing fluid viscosity increases the residence time to some extent by delaying the tear action [26]. This is in agreement with our observations since the reference marketed gels, sodium carboxymethylcellulose and sodium hyaluronate, are more viscous than the other components, and presented a higher ocular residence time with a MRT of 197 and 134 min, respectively. Furthermore, the CD solutions presented a slight viscosity of around 6 mPa.s, which resulted in a significant increase in T_1/2_ and MRT values when compared to BSS. The MRT value for Gel B (112 min) was between the values obtained for CELLUVISC^®^ (197 min), VISMED^®^ (134 min), and DXMa/HPγCD solution (101 min). In addition, the presence of both CMC and HA, associated with higher gel B viscosity, seems to promote ocular remanence. The low MRT value of 67 min obtained in the case of Gel A is rather surprising with respect to the observed HPβCD solution MRT value (118 min). This would merit further investigation since a high variability in the results was observed. Furthermore, sodium hyaluronate, present in Gel A, Gel B, and VISMED^®^, is a shear thinning fluid. Sodium hyaluronate contributes to enhance viscosity while avoiding excessive stress during blinking [27].

The data summarized in Table 4 show that pharmacokinetic parameters such as T_1/2_, MRT, and k are significantly different between each gel and BSS at p < 0.05.The data collected from 3 to 240 min were significantly different between Gels A and B, DXMa (10 mg/mL)/HPβCD (600 mg/mL), DXMa (30 mg/mL)/HPγCD (600 mg/mL), VISMED^®^, and CELLUVISC^®^ at p < 0.05.

### 3.7. Stability

The stability of Gels A and B was assessed using the following parameters: visual inspection, presence or absence of visible particles, DXMa concentration, presence or absence of breakdown products, pH, and osmolality. The study was conducted according to ICH Q1A (R2) methodological guidelines for stability studies [15,40]. A variation of DXMa concentration outside 90–110% intervals of the initial concentration was considered as a sign of a significant DXMa concentration variation. The observed gels must be limpid, of unchanged color, and clear with no visible signs of haziness or precipitation. pH values were considered to be acceptable if they did not vary by more than one pH unit from the initial value.

Gels A and B stayed limpid and there was no appearance of any visible particulate matter, haziness, or gas development. Every Gel A presented a slightly yellowish tinge throughout the study.

**Figure 9:**
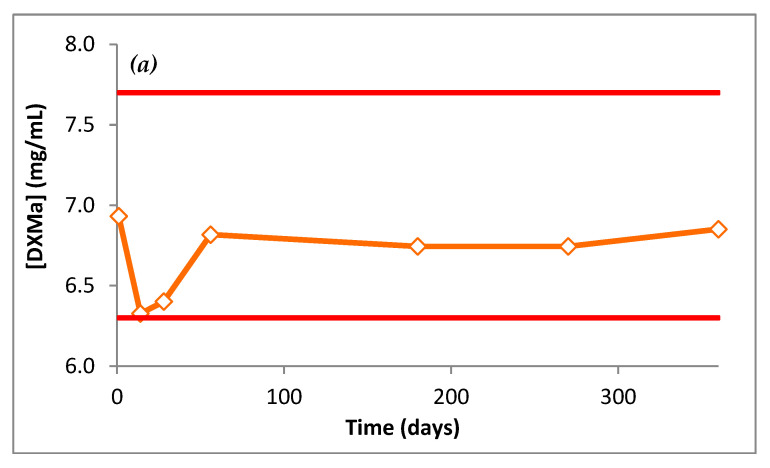

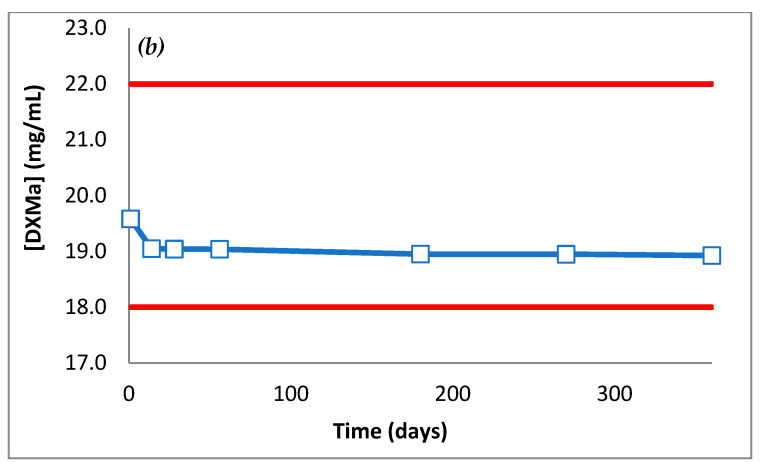
DXMa concentrations (mg/mL) of (**a**) Gel A and (**b**) Gel B as a function of time.

The DXMa concentrations during 12 months are presented in Figure 9. Throughout the dosage times, Gel A and B stored at 25 °C did not vary by more than 10% of the initial concentrations, with low variability as 95% confidence intervals.

For each gel, pH did not vary by more than 0.3 pH units from D0 to M12. pH Gel A was 7.5 and pH Gel B was 7.0. At 12 months, osmolality of Gel A and B had not varied by more than 2.5% of initial osmolality. Both pH and osmolality did not vary during 12 months and stayed within an acceptable physiological range.

Since autoclaving led to DXMa degradation, Gels A and B were sterilized by a validated filtration methodology. The samples of gels conserved at 25 °C in unopened bottles at day 0, 14 days, 30 days and 2, 6, 9, and 12 months did not show any signs of microbiological growth (< 1UFC), meeting the requirements of the 2.6.1 European Pharmacopeia.

## 4. Conclusions and Future Prospects

In conclusion, the data provided in this study demonstrate that the use of hydrogels combined with hydrosoluble cyclodextrins is relatively safe, increases ocular retention, and could act as penetration promoters for DXMa. Indeed, both gels present a good corneal permeation, which was 3.22-fold higher than DEXAFREE^®^ and 4.04-fold higher than MAXIDEX^®^ for Gel B and 1.8-fold higher than DEXAFREE^®^ and 2.5-fold higher than MAXIDEX^®^ for Gel A. Furthermore, they were stable at 25 °C during 12 months after filtration sterilization. These good results have to be confirmed in vivo with pharmacokinetic, efficacy, and tolerance studies.

## Figures and Tables

**Table 1:** Composition of optimized mixed Gels A and B.

Mixed Gels	Components	Quantity (g)
Optimized mixed Gel A	VISMED^®^	0.300
	HPβCD 600 mg/mL with DXMa	0.700
	Optimized mixed Gel A contains 7 mg/g of DXMa and an osmolality of 449 mOsm/kg	
Optimized mixed Gel B	CELLUVISC^®^	0.151
	VISMED^®^	0.085
	HPγCD 600 mg/mL with DXMa	0.764
	Optimized mixed Gel B contains 20 mg/g of DXMa and an osmolality of 425 mOsm/kg	

**Table 2:** Sample concentration levels of DXMa for Gels A and B.

Gels	Level 80%	Level 90%	Level 100%	Level 110%	Level 120%
Gel A (µg/mL)	56	63	70	77	84
Gel B (µg/mL)	160	180	200	220	240

**Table 3:** Prediction model inspired by the short time exposure according to the Organization for Economic Co-operation and Development (OECD) guidelines [32].

Cell Viability		UN GHS Classification	Applicability	DXMa Formulation Vehicles
At 5%	At 0.05%			
>70%	>70%	No category	No serious damage nor eye irritation effect	Gel B
				HPγCD aqueous solution (600 mg/mL)
≤70%	>70%	No prediction can be made	No prediction can be made, eventual eye irritation	Gel A
				HPβCD aqueous solution (600 mg/mL)
≤70%	≤70%	Category 1	Serious eye damage	None

**Table 4:** Ocular biopermanence parameters measured in vivo for Gels A and B, DXMa (10 mg/mL)/HPβCD (600 mg/mL), DXMa (30 mg/mL)/HPγCD (600 mg/mL), VISMED^®^, and CELLUVISC^®^ versus Balanced Salt Solution (BSS).

Components	Viscosity at 35 °C (mPa.s)	k (min^−1^)	T_1/2_ (min)	MRT (min)	R^2^
CELLUVISC^®^	167–260	0.007 ± 0.003	136.5 ± 95.5	196.9 ± 137.8	0.9738
VISMED^®^	16.8	0.008 ± 0.003	92.7 ± 26.7	133.7 ± 38.5	0.9404
Gel B	18.6	0.0096 ± 0.036	77.4 ± 28.8	111.6 ± 41.5	0.9837
Gel A	13.2	0.015 ± 0.002	46.6 ± 4.8	67.2 ± 6.9	0.9365
DXMa/HPβCD	6.4	0.015 ± 0.014	81.7± 59.0	117.9± 85.2	0.9866
(10 mg/mL/600 mg/mL)					
DXMa/HPγCD	6.5	0.11 ± 0.003	70.2 ± 21.9	101.3 ± 31.6	0.9697
(30 mg/mL/600 mg/mL)					
BSS	1.5	0.046 ± 0.015	16.0 ± 5.2	23.1 ± 7.6	0.9965

## Supplementary Materials

The following are available online at https://www.mdpi.com/1999-4923/12/8/717/s1, Figure S1. A, B, C and D show the chromatogram of DXMa for Gel A diluted to 70 µg/mL, DXMa in Gel A diluted to 70 µg/mL and DXMa for Gel B diluted to 200 µg/mL and DXMa in Gel B diluted to 200 µg/mL obtained with the chromatographic methods used. Figure S2. Calibration curves for DXMa in Gel A and Gel B (3 days/5 levels a day). Figure S3. Chromatograms obtained for DXMa in Gel A after applying different stress conditions. (a) No stress, (b) HCl 0.5 N at 80 °C during 1 h, (c) NaOH 0.5 N at 80 °C during 1 h, (d) H_2_O_2_ 3% at 80 °C during 4 h and (e) UV light for 6 h. Figure S4. Chromatograms obtained for DXMa in Gel B after applying different stress conditions. (a) No stress, (b) HCl 0.5 N at 80 °C during 1 h, (c) NaOH 0.5 N at 80 °C during 1 h, (d) H_2_O_2_ 3% at 80 °C during 4 h and (e) UV light for 6 h. Figure S5. Chromatograms before and after autoclaving Gel A. Figure S6. Chromatograms before and after autoclaving Gel B. Figure S7. Amplitude sweep test performed with Gel B at 0.1, 1 and 10 Hz at 35 °C. Table S1. Calibration curves of DXMa in Gel A and Gel B. Table S2. Limit of detection and quantification for Gels A and B.
