# Supplementary material for: Biopharmaceutical Assessment of Dexamethasone Acetate-Based Hydrogels Combining Hydroxypropyl Cyclodextrins and Polysaccharides for Ocular Delivery
Source: Pharmaceutics. 2020 Jul 30;12(8):717. doi: 10.3390/pharmaceutics12080717 (PMC7464375; doi:10.3390/pharmaceutics12080717)
Supplement: Supplementary file 1 [file pharmaceutics-12-00717-s001.pdf]

Supplementary Materials: Biopharmaceutical Assessment of Dexamethasone Acetate-Based Hydrogels Combining Hydroxypropyl Cyclodextrins and Polysaccharides for Ocular Delivery

Roseline Mazet, Xurxo García Otero, Luc Choisnard, Denis Wouessidjewe, Vincent Verdoot, Frédéric Bossard, Victoria Díaz Tomé, Véronique Blanc-Marquis, Francisco-Javier Otero-Espinar, Anxo Fernandez-Ferreiro * and Annabelle Gèze *

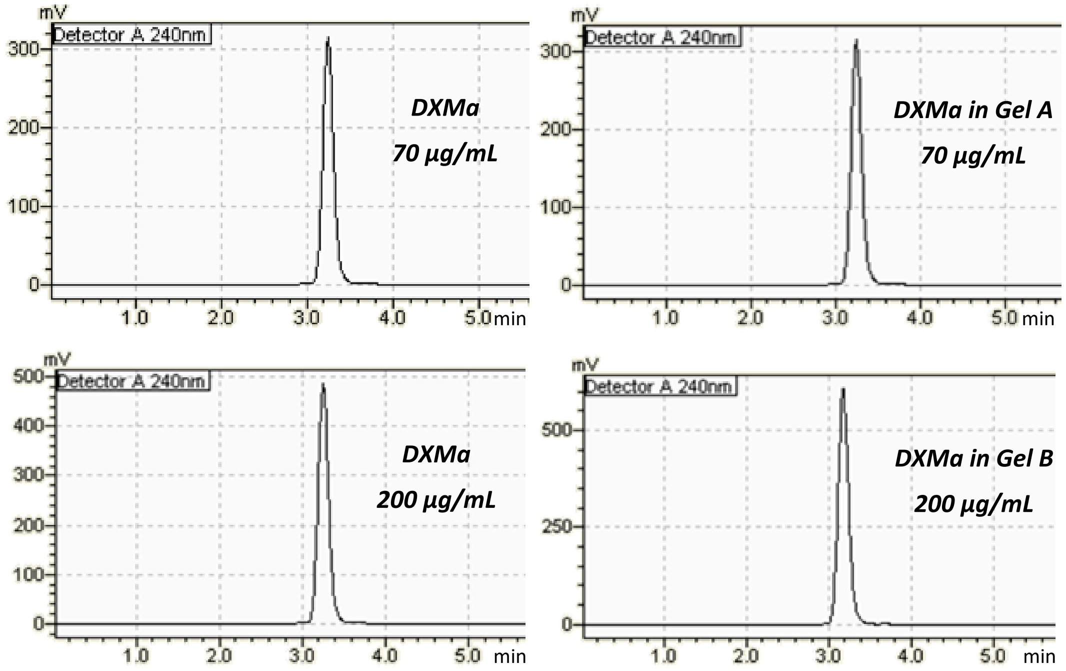

Figure S1. A, B, C and D show the chromatogram of DXMa for Gel A diluted to 70 µg/mL , DXMa in Gel A diluted to 70 µg/mL and DXMa for Gel B diluted to 200 µg/mL and DXMa in Gel B diluted to 200 µg/mL obtained with the chromatographic methods used.

Method validation studies

The RP-HPLC method used to analyze the DXMa in Gels A and B was validated according to current ICH Q2(R1) [13].The performed validation tests proved the suitability of the method for its intended purposes. Validation tests including specificity, linearity and range parameter, accuracy, precision, LOQ, LOD. Original validation data are reported in supplementary material.

Linearity

The linearity was required to demonstrate that the detector response is directly proportional to the analyte concentration over a specific range. The evaluation of calibration curves was made with five different known concentrations of DXMa (80, 90, 100, 110 and 120% of the specification level), daily injected in duplicates, three days during.

Original data for validation of analytical dosage methods

Data concerning linearity of DXMa					
**Group i (Level)**	**Assay j (day)**	**Concentration x_{ij}(µg/mL)**	**AUC $y_{ij\,1}$**	**AUC $y_{ij\,2}$**	**Average AUC y_{ij}**
**1**	**1**	56,090	1588077	1588124	1588101
**1**	**2**	56,090	1547002	1546820	1546911
**1**	**3**	56,09	1520954	1539070	1530012
**2**	**1**	63,101	1774759	1770105	1772432
**2**	**2**	63,101	1735297	1741892	1738595
**2**	**3**	63,101	1787189	1781234	1784212
**3**	**1**	70,112	1937731	1938898	1938315
**3**	**2**	70,112	1924412	1924942	1924677
**3**	**3**	70,112	1948512	1946367	1947440
**4**	**1**	77,120	2130570	2141625	2136098
**4**	**2**	77,120	2128707	2128697	2128702
**4**	**3**	77,120	2125759	2138035	2131897
**5**	**1**	84,130	2310641	2313094	2311868
**5**	**2**	84,130	2324057	2325693	2324875
**5**	**3**	84,130	2313642	2316523	2315083

Data concerning linearity of DXMa in Gel A					
**Group i (Level)**	**Assay j (day)**	**Concentration x_{ij}(μg/mL)**	**AUC $y_{ij\,1}$**	**AUC $y_{ij\,2}$**	**Average AUC y_{ij}**
**1**	**1**	56,000	1553272	1552935	1553104
**1**	**2**	56,000	1551302	1543942	1547622
**1**	**3**	56,000	1566062	1555866	1560964
**2**	**1**	63,100	1772311	1774311	1773311
**2**	**2**	63,100	1777449	1783165	1780307
**2**	**3**	63,100	1743901	1743134	1743518
**3**	**1**	70,112	1979040	1980674	1979857
**3**	**2**	70,112	1959011	1959320	1959166
**3**	**3**	70,112	1942446	1947436	1944941
**4**	**1**	77,120	2102508	2128252	2115380
**4**	**2**	77,120	2094166	2074850	2084508
**4**	**3**	77,120	2089469	2102097	2095783
**5**	**1**	84,130	2325019	2323786	2324403
**5**	**2**	84,130	2324102	2325355	2324729
**5**	**3**	84,130	2324567	2335678	2330123

Data concerning intermediate fidelity of DXMa in Gel A			
**Group i (Level)**	**Assay j (day)**	**Concentration x_{ij}(μg/mL)**	**AUC $y_{ij\,1}$**
**1**	**1**	70,112	1937731
**1**	**2**	70,112	1938898
**1**	**3**	70,112	1944643
**1**	**4**	70,112	1944588
**1**	**5**	70,112	1945897
**1**	**6**	70,112	1934567
**2**	**1**	70,112	1924412
**2**	**2**	70,112	1924942
**2**	**3**	70,112	1930883
**2**	**4**	70,112	1933463
**2**	**5**	70,112	1934576
**2**	**6**	70,112	1945632
**3**	**1**	70,112	1948512
**3**	**2**	70,112	1946367
**3**	**3**	70,112	1948860
**3**	**4**	70,112	1939592
**3**	**5**	70,112	1945631
**3**	**6**	70,112	1949087

Data concerning linearity of DXMa					
**Group i (Level)**	**Assay j (day)**	**Concentration x_{ij}(μg/mL)**	**AUC $y_{ij\,1}$**	**AUC $y_{ij\,2}$**	**Average AUC y_{ij}**
**1**	**1**	160,260	4390650	4388550	4389600
**1**	**2**	160,260	4376427	4374207	4375317
**1**	**3**	160,260	4331524	4407674	4369599
**2**	**1**	180,290	4976139	4976872	4976506
**2**	**2**	180,290	5030549	5033908	5032229
**2**	**3**	180,290	4978558	4981948	4980253
**3**	**1**	200,320	5654170	5690387	5672279
**3**	**2**	200,320	5613164	5614173	5613669
**3**	**3**	200,320	5604309	5686397	5645353
**4**	**1**	220,040	6227256	6227115	6227186
**4**	**2**	220,040	6269243	6277131	6273187
**4**	**3**	220,040	6234668	6235969	6235319
**5**	**1**	240,380	6804130	6807503	6805817
**5**	**2**	240,380	6839372	6839752	6839562
**5**	**3**	240,380	6853296	6848154	6850725

Data concerning linearity of DXMa in Gel B					
**Group i (Level)**	**Assay j (day)**	**Concentration x_{ij}(μg/mL)**	**AUC $y_{ij\,1}$**	**AUC $y_{ij\,2}$**	**Average AUC y_{ij}**
**1**	**1**	159,530	4437552	4383902	4410727
**1**	**2**	159,530	4440132	4374644	4407388
**1**	**3**	159,530	4390134	4381900	4386017
**2**	**1**	179,990	4935648	4917118	4926383
**2**	**2**	179,990	4921226	4885186	4903206
**2**	**3**	179,990	4900604	4878454	4889529
**3**	**1**	199,860	5664654	5658696	5661675
**3**	**2**	199,860	5665840	5665940	5665890
**3**	**3**	199,860	5670640	5688068	5679354
**4**	**A**	220,030	6239162	6228308	6233735
**4**	**2**	220,030	6246426	6234646	6240536
**4**	**3**	220,030	6222518	6225324	6223921
**5**	**1**	240,020	6803372	6806470	6804921
**5**	**2**	240,020	6813104	6808300	6810702
**5**	**3**	240,020	6826300	6826300	6826300

Data concerning intermediate fidelity of DXMa in Gel B			
**Group i (Level)**	**Assay j (day)**	**Concentration x_{ij}(μg/mL)**	**AUC $y_{ij\,1}$**
**1**	**1**	200,320	5654170
**1**	**2**	200,320	5690387
**1**	**3**	200,320	5679719
**1**	**4**	200,320	5674248
**1**	**5**	200,320	5687689
**1**	**6**	200,320	5663442
**2**	**1**	200,320	5613164
**2**	**2**	200,320	5614173
**2**	**3**	200,320	5636475
**2**	**4**	200,320	5631799
**2**	**5**	200,320	5658997
**2**	**6**	200,320	5648976
**3**	**1**	200,320	5604309
**3**	**2**	200,320	5686397
**3**	**3**	200,320	5684617
**3**	**4**	200,320	5654929
**3**	**5**	200,320	5642356
**3**	**6**	200,320	5678493

The standard calibration curves plotted the obtained mean peak area as a function of the concentration of DXMa are reported in **Error! Reference source not found.**2 for both Gels A and B.

The regression parameters of the lines are reported in Table S1.

Table S1. Calibration curves of DXMa in Gel A and Gel B.

Gel	Range of linearity (µg/mL)	Slope	intercept	Correlation coefficient R^2
Gel A	56 - 84	26986	49280	0.996
Gel B	160 - 240	30783	-545504	0.999

Slopes were significantly different from zero (p-value < 5%) and interceptions were not significantly different from zero (p-value > 5%). The determination coefficient (R^2) value was found to be > 0.99. Hence, the method has linear response over the performed concentration range.

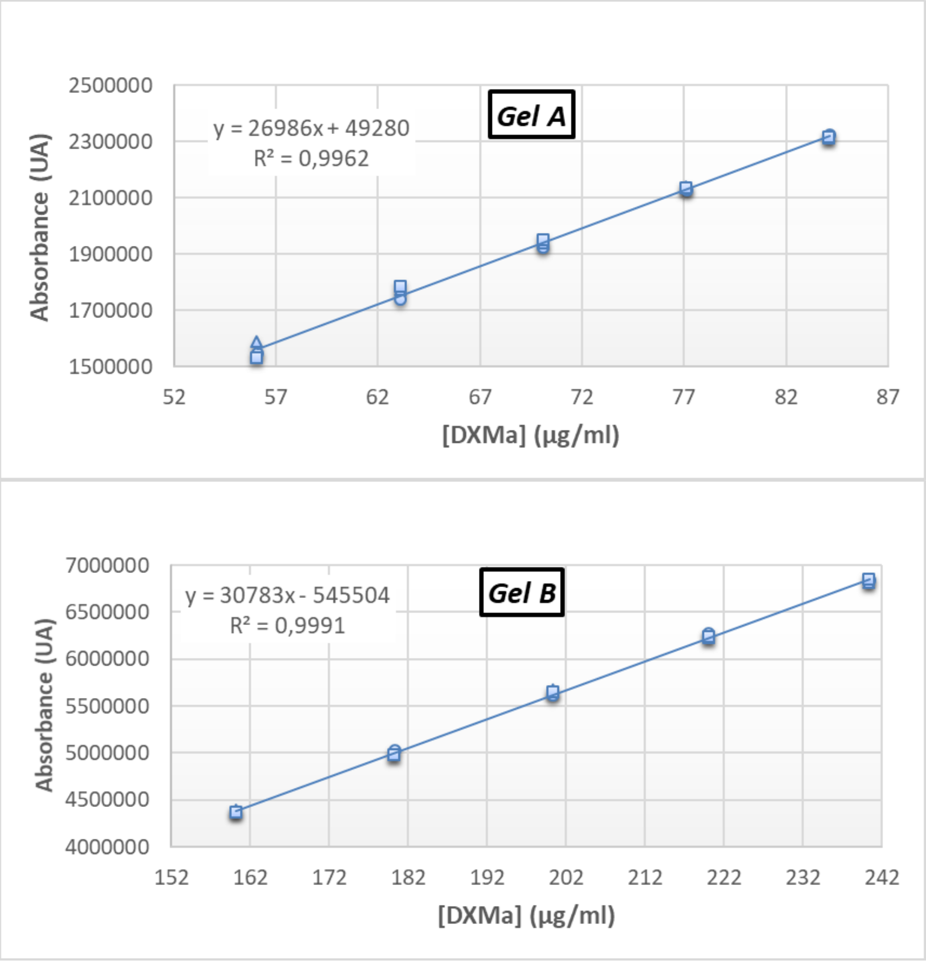

Figure S2. Calibration curves for DXMa in Gel A and Gel B (3 days/5 levels a day).

Accuracy (Bias %)

The accuracy studies were performed to verify the closeness of the agreement between the expected and the determined values. The DXMa concentration spiked in Gels A or B were determined using a linear regression. The accuracy was evaluated by calculating first the percentage recovery and then the percentage of relative standard deviation (RSD) of recovery. The recovery results obtained from the five standards of calibration levels were between 98.48 and 101.07% for DXMa in Gel A and between 98.40 and 101.01% for DXMa in Gel B. The values are within the limit of acceptance (95–105%). The RSD (%) of all five levels were 1.31% for DXMa in Gel A and 0.97% for DXMa in Gel B. The results were lower than the limit of acceptance (2%), indicating that the method is accurate.

Specificity

Specificity was examined by analyzing only the excipients of each gel (Gel A or B without DXMa). The absence of interference with DXMa was demonstrated (chromatogram not shown). In complement, to prove the specificity of the method, the degradation studies under relevant stress conditions were also performed and degradation products were observed after stress treatment (Figures S3 and S4).

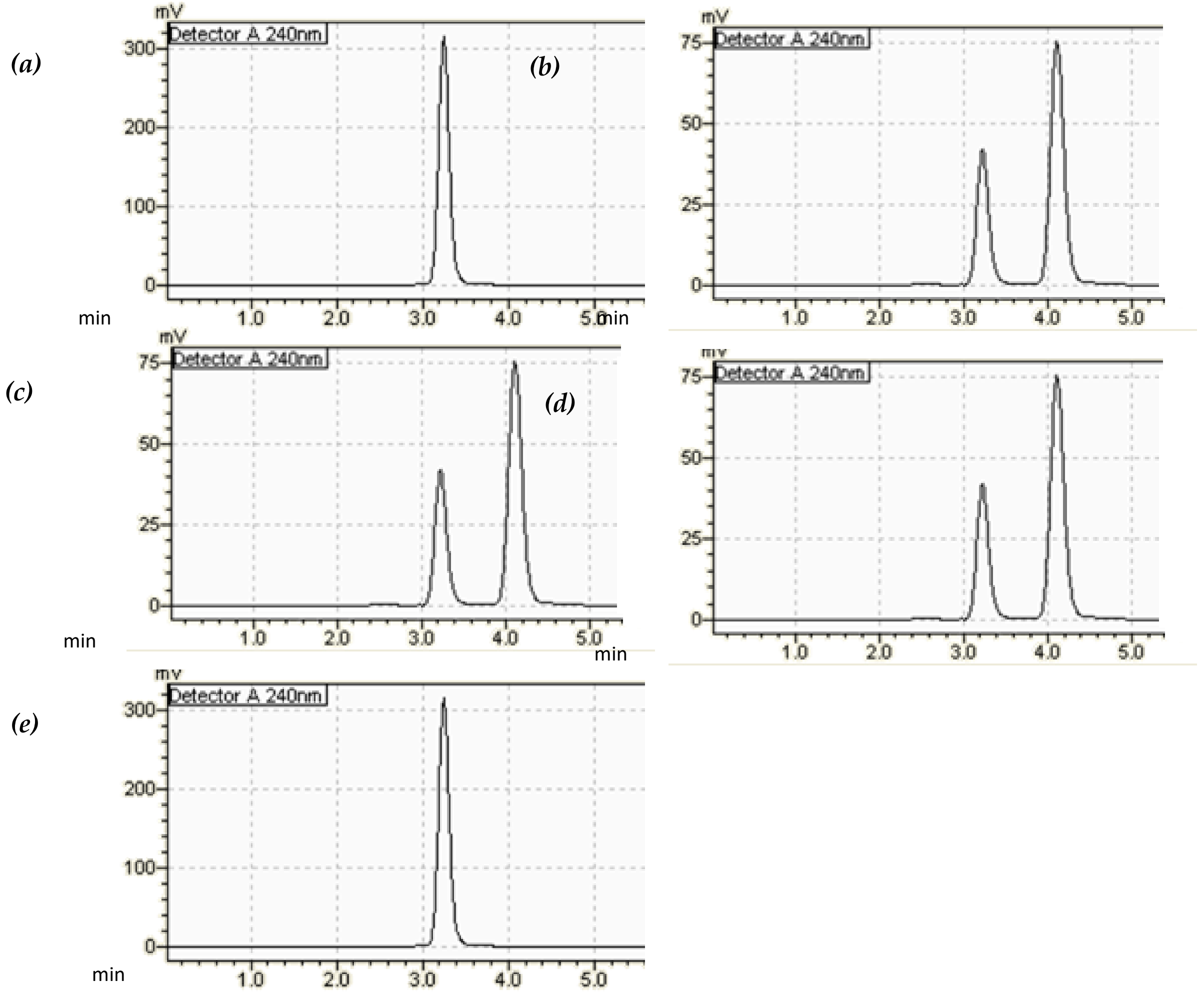

Figure S3. Chromatograms obtained for DXMa in Gel A after applying different stress conditions. (a) No stress, (b) HCl 0.5 N at 80 °C during 1 h, (c) NaOH 0.5 N at 80 °C during 1 h, (d) H_2O_2 3 % at 80 °C during 4 h and (e) UV light for 6 h.

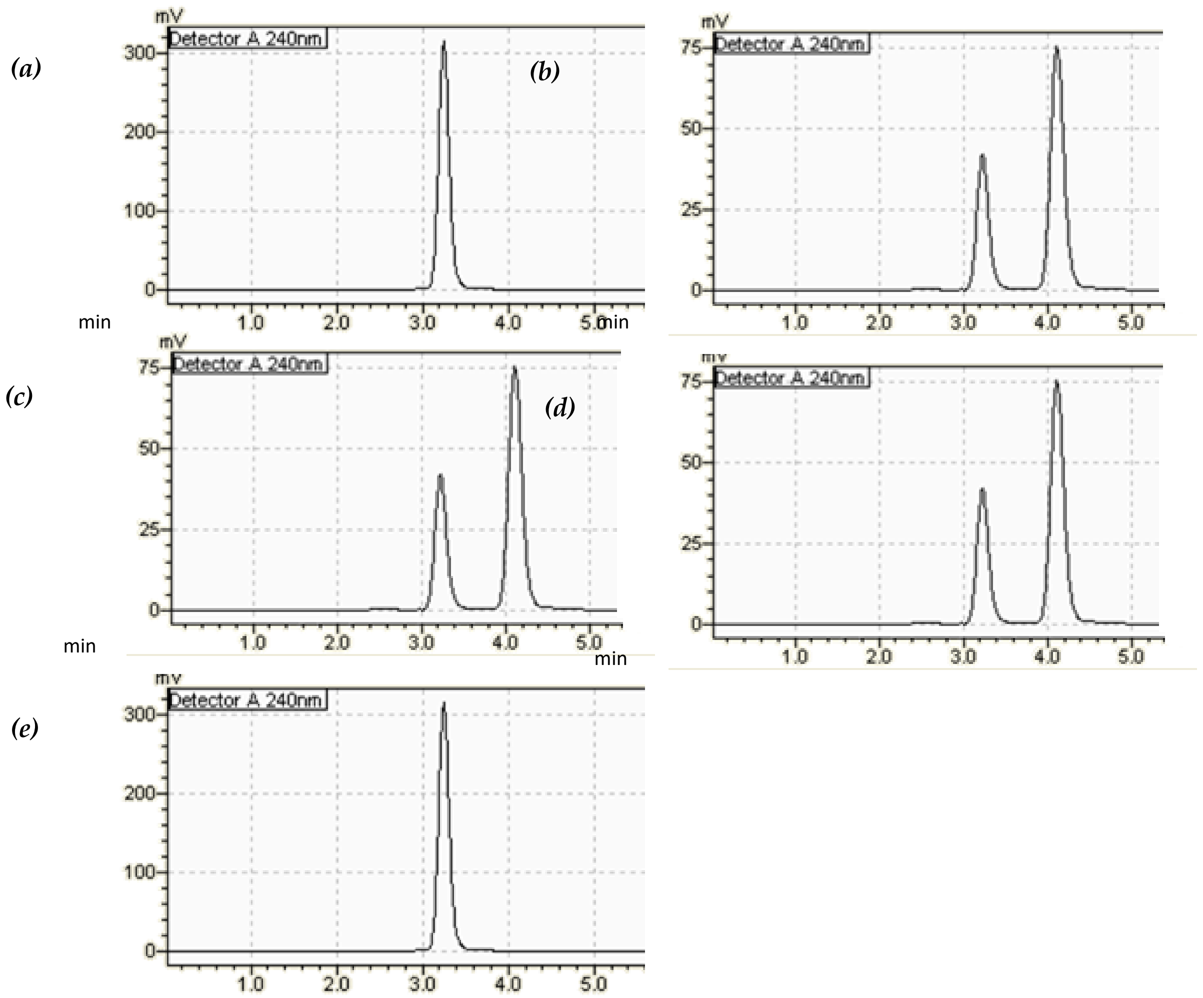

Figure S4. Chromatograms obtained for DXMa in Gel B after applying different stress conditions. (a) No stress, (b) HCl 0.5 N at 80 °C during 1 h, (c) NaOH 0.5 N at 80 °C during 1 h, (d) H_2O_2 3 % at 80 °C during 4 h and (e) UV light for 6 h.

None of the observed peaks interfered with the DXMa peak in terms of retention time (resolution greater than 1.5). The used methods are therefore capable of identifying degradation products separately from DXMa. It should be noted that at this stage, we did quantify these degradation products.

Precision

Synthetic blend solutions representing 100 % of the target concentration of the method were used. The precision parameter was evaluated by performing both repeatability (intra-day variability) and intermediate precision (inter-day variability).

The repeatability characterizes the reproducibility of a given analytical procedure for the same sample preparation, as performed by the same analyst using the same instrument during a relatively short period time (intra-day). The repeatability was demonstrated by preparing six sample solutions (100%) measured by HPLC and calculating the relative percentage of standard deviation (RSD). For both formulations, the repeatability RSD values were 0.29% (Gel A) and 0.36% (Gel B). The RSD (%) values for intra-day are found to be < 2%, which were considered acceptable.

The intermediate precision characterizes the reproducibility of results obtained in the same laboratory during a prolonged period. It was established by preparing six assay sample solutions similar to repeatability (level 100%) injected into a HPLC system as per proposed method on 3 different days. The RSD (%) of assay results was calculated. The intermediate precision results are 0.44% for Gel A and 0.55% for Gel B. The RSD (%) values for inter-day precision were found to be

lower than 2%, which indicates that method is also reproducible. The method was considered to be precise.

Limit of detection and limit of quantification

Detection and quantification limits are the lowest detectable and quantifiable concentration that a method can achieve (Table S2). As per ICH guideline, the LOD and LOQ were determined based on the standard deviation of the response (σ) and the slope (s) in accordance with the equations: LOD = 3.3 × σ/s and LOQ = 10 x σ/S.

Table S2. Limit of detection and quantification for Gels A and B**.**

Gel	LOD (μg/mL)	LOQ (μg/mL)
Gel A	2.16	6.55
Gel B	3.06	9.26

In conclusion, the chromatographic method described was validated for quantitative assay determination of DXMa in Gels A and B as per ICH Q1A (R2) guideline.

The developed method is specific, accurate, precise, and reproducible. All the degradation products formed during stress conditions were well separated from the DXMa peak demonstrating that the developed method was specific. The method, according to international guidelines, can be used to determine DXMa content over time since no interference with degradation products was observed.

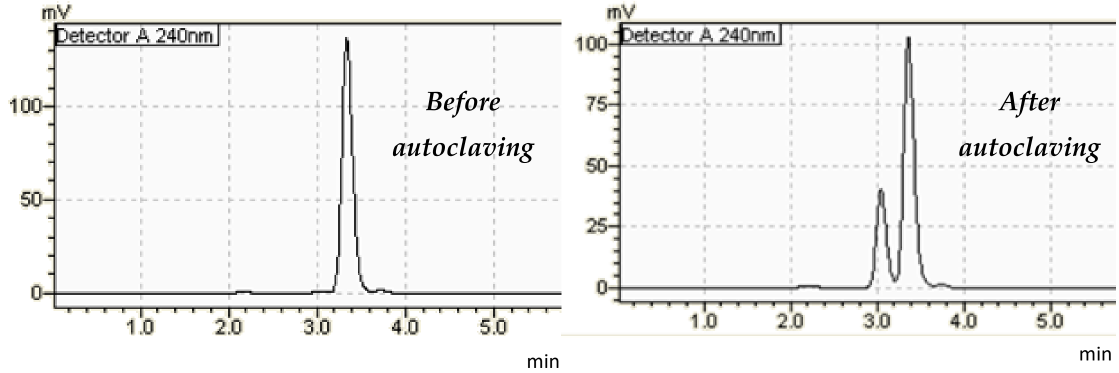

Figure S5. Chromatograms before and after autoclaving Gel A.

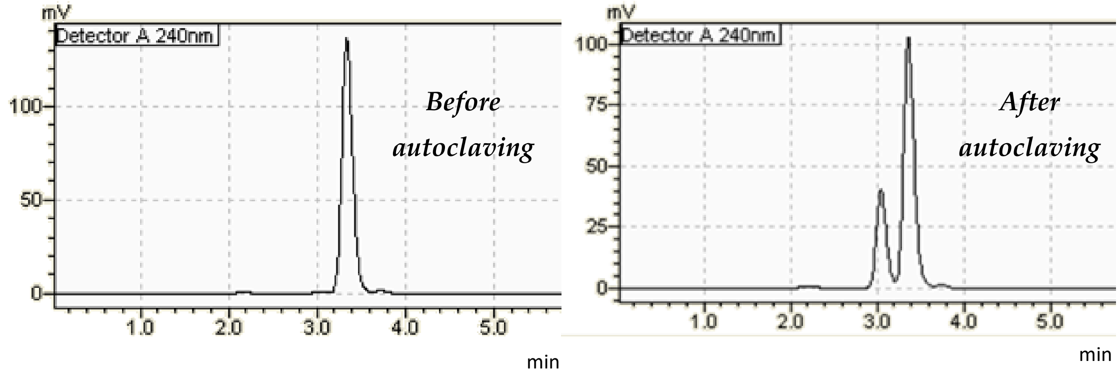

Figure S6. Chromatograms before and after autoclaving Gel B.

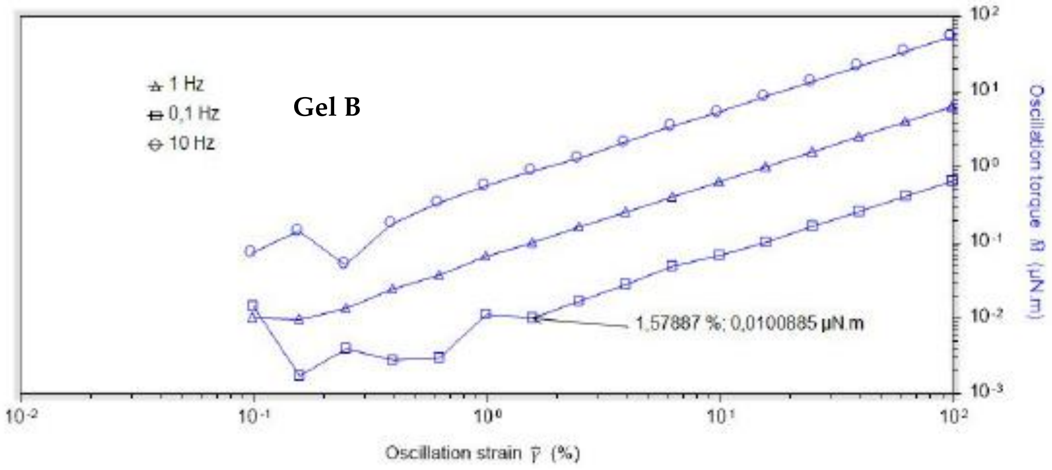

Figure S7. Amplitude sweep test performed with Gel B at 0.1, 1 and 10 Hz at 35 °C**.**
